# Inadvertent placement of thoracic stent grafts in false lumen during aortic dissection surgery – unicentric analysis and systematic review

**DOI:** 10.1097/JS9.0000000000003028

**Published:** 2025-07-24

**Authors:** Hsiu-Ming Lee, Ying Sheng Li, Mingli Levin Li

**Affiliations:** a Department of Surgery, Chang Gung Memorial Hospital at Linkou, Chang Gung University, Taoyuan, Taiwan; b Division of Thoracic and Cardiovascular Surgery, Department of Surgery, Chang Gung Memorial Hospital at Linkou, Chang Gung University, Taoyuan, Taiwan; c Cardiovascular Surgery, China Medical University Hospital, Taichung City, Taiwan

**Keywords:** aortic dissection, FET, frozen elephant trunk, intervention, intravascular ultrasound, IVUS, misplacement, prognosis, TEE, TEVAR, thoracic endovascular aortic repair, transesophageal echocardiogram

## Abstract

**Background:**

The aim of this retrospective study and systematic review was to examine the causes, management strategies, and outcomes of thoracic endovascular aortic repair (TEVAR) stent graft misplacement in the false lumen (FL). Accordingly, we analyzed cases from China Medical University Hospital along with data from published articles.

**Materials and Methods:**

This single-center retrospective study analyzed six TEVAR stent graft misplacement cases among 1227 patients who had an aortic dissection and underwent TEVAR at China Medical University Hospital (2011–2024). Furthermore, a systematic search of PubMed, Embase, Web of Science, and Cochrane CENTRAL and ClinicalTrials.gov for relevant studies was performed. Outcome data on symptoms, clinical outcomes, placement method, management strategies, treatment quality, potential causes of misplacement, and factors related to oversight were independently extracted by two reviewers in a standardized manner.

**Results:**

A total of 35 cases from 23 studies, including 6 from our institution, were reviewed. TEVAR misplacement predominantly occurred in type A dissections (62.9%) and in cases of anterogradely placed TEVAR stents (68.6%). Complications, mainly visceral malperfusion (48.6%), were reported in 77.1% of the cases. Intraoperative misplacement was more accurately detected by transesophageal echocardiogram (TEE) or intravascular ultrasound (IVUS) than by aortography alone (*P* < 0.001). Endovascular retrograde stent extension with or without septal fenestration was linked to improved survival (*P* = 0.018). Early symptom onset within 3 days and delayed treatment increased mortality (*P* = 0.029). Overall mortality was 28.6%, mainly due to multiorgan failure (80%).

**Conclusion:**

Although rare, accidental TEVAR stent graft placement in the FL markedly increases mortality. Despite seemingly acceptable survival rates, challenges in diagnosis and the potential for publication bias may lead to misleading conclusions. Clinicians must immediately administer comprehensive aortography, TEE, IVUS, or intraoperative computed tomography when they suspect an instance of accidental placement to ensure timely intervention.

## Introduction

The development of the “frozen elephant trunk” (FET) procedure, which combines thoracic endovascular aortic repair (TEVAR) with open arch surgery, has led to improved outcomes in the treatment of complex aortic pathologies^[1,2]^. The FET procedure involves the use of a hybrid prosthesis that combines a Dacron graft with a distally positioned endovascular stent graft made of nitinol or steel; this prosthesis is placed at the distal aortic arch to the proximal descending aorta^[3]^. This technique has been further refined through integration with total arch replacement^[4]^. The FET technique stabilizes the descending aorta, seals the entry tear, and depressurizes the false lumen (FL)^[5]^. Although complications such as distal stent graft–induced new entry (dSINE) can occur, TEVAR has demonstrated favorable outcomes in restoring blood flow and preventing aortic enlargement or rupture, rendering it a widely adopted technique for aortic dissection^[6,7]^.

**HIGHLIGHTS**

- Thoracic endovascular aortic repair (TEVAR) stent misplacement into the false lumen is rare but can be life-threatening if undetected or poorly managed.
- TEVAR stent misplacement predominantly occurred in type A aortic dissection (62.9%) and anterogradely placement (68.6%) cases.
- Transesophageal echocardiogram or intravascular ultrasound had higher diagnostic accuracy compared with aortography alone (*P* < 0.001).
- Early intraoperative reintervention, particularly endovascular retrograde stent extension with or without septal fenestration, reduced mortality (*P* = 0.018), whereas delayed treatment and early symptom onset within 3 days increased mortality (*P* = 0.029).

Correct placement of the TEVAR device in the true lumen (TL) is essential for proper blood flow. However, misplacement of the TEVAR device in the FL can cause severe complications such as increased FL pressure, malperfusion, and aortic rupture. Intraoperative misplacement may not always be identified, even with imaging, leading to misdiagnosis, delayed treatment, and even unexplained death. This complication can result in visceral, spinal, or lower limb malperfusion; shock; or severe suture-line bleeding, with a high risk of mortality. Nonetheless, these severe complications are potentially treatable through open or percutaneous rescue techniques. However, case reports and comprehensive investigations of such complications are scant because of their rarity. Accordingly, we conducted a 14-year retrospective survey at our hospital and a systematic review to evaluate previous cases, management strategies, and outcomes related to TEVAR device misplacement in the FL.

In line with the TITAN 2025 guidelines^[8]^, ChatGPT (OpenAI and GPT-4) was used solely to improve grammar and language clarity. No AI was involved in data analysis, interpretation, conceptual development, figure generation, or content generation. No patient data were provided to the AI, and all inputs complied with data protection standards. The authors have critically reviewed all AI outputs and remain fully responsible for the manuscript’s integrity and accuracy.

## Methods

### Study protocol and registration

Our retrospective, single-center observational study analyzed all cases of TEVAR device misplacement among 1227 patients with aortic dissection who underwent TEVAR at our hospital between 2011 and 2024. The study complied with the principles of the Declaration of Helsinki and was approved by the institutional review board of our hospital; the need for patient consent was waived (Project ID: 113-REC1-099). Our systematic review adheres to the Preferred Reporting Items for Systematic Reviews and Meta-Analyses (PRISMA) statement and the Assessing the Methodological Quality of Systematic Reviews (AMSTAR) guidelines. It has been registered in the PROSPERO database^[9,10]^.

### Data sources and search strategy

Two reviewers independently developed and conducted the search strategy using PubMed, Cochrane CENTRAL, Embase, Web of Science, and ClinicalTrials.gov. They searched for studies published between 1 January 1990 and 31 May 2025. Titles, abstracts, and full texts were screened, with disagreements resolved by a third reviewer. Backward and forward reference searches were conducted through May 2025. Supplemental Digital Content Table S2, available at: http://links.lww.com/JS9/E774, provides the details of the search strategy.

### Inclusion and exclusion criteria

The inclusion criteria for the studies were as follows: (1) including patients with aortic disease undergoing FET or TEVAR; (2) including patients whose thoracic stent grafts were placed in the FL; (3) being a case report, case series, letter, correspondence, or commentary; and (4) being published in English. The exclusion criteria were as follows: (1) being a meeting abstract with no data, (2) being a commentary with no data, (3) including cases of intentional FL stent graft placement^[11,12]^, (4) including cases of stent graft migration due to dSINE or kinking,^[13–15]^ (5) including cases of thoracic stent grafts not classified as FET (e.g., traditional elephant trunk or bare metal stents)^[16–22]^, (6) not providing case descriptions, or (7) not providing full text. Our primary outcome of interest was the intraoperative detection rate of TEVAR misplacement into the FL. Our secondary outcomes were patient prognosis, overall mortality, cause of death, and the effectiveness of management strategies.

### Study selection

**Figure 1.**
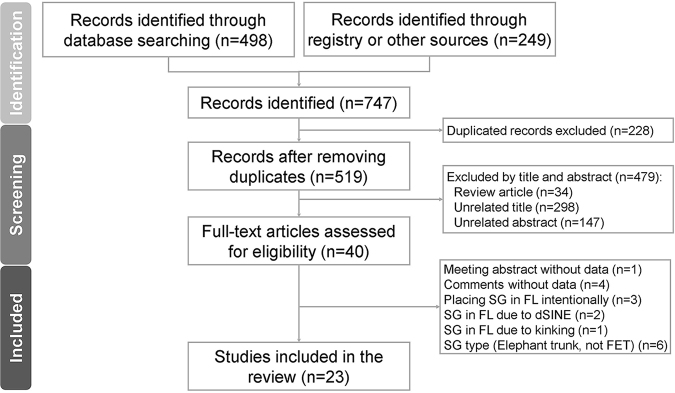
PRISMA flowchart outlining study selection process for review.

After compiling and de-duplicating data, the two reviewers independently screened prospective articles in two stages. First, they reviewed titles and abstracts and excluded those that did not meet the inclusion criteria or were not accessible; however, they retained abstracts that did not provide sufficient information for further assessment. Second, they independently assessed full-text articles and excluded those that did not meet the inclusion criteria. Disagreements were resolved through discussion with a third reviewer. The study selection process is outlined in Figure 1.

### Data extraction

The two reviewers independently extracted data using a standardized template, collecting information on (1) patient demographics (age, sex, and aortic dissection type); (2) initial stent graft placement details and TL detection techniques; (3) stent displacement symptoms; (4) misplacement detection methods and timing; (5) potential misplacement mechanisms; (6) reintervention details (timing, method, and TL locating approach); and (7) outcomes (prognosis, follow-up duration, and cause of death).

### Methodological quality appraisal

Case reports are prone to bias. Therefore, standardized tools have been developed to assess their methodological quality in systematic reviews^[23]^. In this study, the two reviewers independently evaluated case quality using a risk-of-bias assessment tool adapted from Murad *et al*^[23,24]^, categorizing each study as having a low, moderate, or high risk of bias.

### Data analysis

The collected demographic and clinical data were summarized using descriptive statistics. Specifically, continuous variables are presented as means with standard deviations, and categorical variables are presented as frequencies and percentages. Univariate Cox proportional hazards models were used to identify significant factors associated with patient outcomes, which were included in multivariate models to assess predictors while controlling for confounding factors. Kaplan–Meier curves were used to compare outcomes based on intervention timing. To reduce bias, three untreated cases and two cases with erroneous stent graft elongation into the FL were excluded. We also used a chi-squared test to compare intraoperative detection rates with or without intravascular ultrasound (IVUS) or transesophageal echocardiogram (TEE).

## Results

### Study characteristic and patient demographics

Figure 1 illustrates the PRISMA flowchart for study selection. Initially, 747 references were identified, with 228 duplicates removed. After titles and abstracts were screened, 479 articles were excluded. The remaining 40 underwent full-text review, leading to 17 exclusions, including 4 commentaries without original data, 1 meeting abstract without data, and 3 case reports where stent grafts were intentionally placed in the FL. Additionally, two reports attributed the stent’s presence in the FL to dSINE, and one cited kinking, whereas six used traditional elephant trunk or bare metal stents instead of the FET. Finally, 23 articles covering 29 patients were included. Furthermore, a retrospective analysis at our institution from February 2011 to September 2024 identified six additional cases, yielding 35 cases in total (Supplemental Digital Content, Table S1, available at: http://links.lww.com/JS9/E773). Of these, 24 involved male patients (68.6%), with a mean age of 54.4 years. Type A aortic dissection (TAAD) was present in 22 cases (62.9%), and type B aortic dissection was present in 12 cases (34.3%), including 3 with concurrent arch aneurysm or non-A non-B dissection. The FET was placed antegradely in 24 cases (68.6%) and retrogradely in 11 (31.4%). Data on sex, age, and dissection type were not reported in six (17.1%), five (14.3%), and one (2.9%) cases, respectively (Table 1).

**Table 1:** Patient characteristics in cases of inadvertent stent graft placement in the false lumen

Characteristics	Patients (*n* = 35)
Sex, *n* (%)	
Male	24 (68.6%)
Female	5 (14.3%)
Unspecified	6 (17.1%)
Age (years)	54.4 ± 14.7
AD type	
TAAD	22 (62.9%)
TBAD	12 (34.3%)
Unspecified	1 (2.9%)
Placement method	
Antegrade	24 (68.6%)
Retrograde	11 (31.4%)
Detection TL/FL method	
Angiography alone	30 (85.7%)
Intra-OP combines IVUS or TEE	5 (14.3%)
Detection misplacement method	
Intra-OP IVUS or TEE	5 (14.3%)
Intra-OP angiography	5 (14.3%)
Post-OP CT	20 (57.1%)
Post-OP IVUS	1 (2.9%)
Undetected	3 (8.6%)
Unspecified	1 (2.9%)
Symptoms	
Visceral malperfusion	17 (48.6%)
Unstable hemodynamics	8 (22.9%)
Active bleeding	5 (14.3%)
Abdominal pain	4 (11.4%)
Asymptomatic	3 (8.6%)
Upper limb hypertension	2 (5.7%)
Aortic dilatation	1 (2.9%)
Aortic rupture	1 (2.9%)
Chest pain	1 (2.9%)
Low extremities numbness	1 (2.9%)
Unspecified	5 (14.3%)
Reintervention time	
Intra-OP	9 (25.7%)
Post-OP (≤3 days)	15 (42.9%)
Post-OP (>3 days)	8 (22.9%)
None	3 (8.6%)
Outcome	
Alive	24 (68.6%)
Expired	10 (28.6%)
Unspecified	1 (2.9%)
Follow-up duration	
N/A (expired)	10 (28.6%)
≤1 month	13 (37.1%)
>1 month	12 (34.3%)

### Clinical characteristics of patients with TEVAR device misplacement

#### Placement techniques and misplacement causes

Among 35 patients, 24 (68.6%) underwent antegrade placement under direct vision, whereas 11 (31.4%) underwent retrograde placement using guidewires. These findings suggest that antegrade placement carries a risk of FL misplacement because of the absence of guidewire navigation and the occasional difficulty in distinguishing the TL from the FL visually. Although pigtail catheters reduce crossing reentry tears during antegrade placement, they do not guarantee appropriate all-the-way TL placement. Among 24 patients undergoing antegrade FET placement, 15 (62.5%) involved FL reentry, possibly caused by unnoticed distal tears, a small TL size, or acute angulation between the distal arch and the descending aorta, which could potentially lead to new artificial intimal tears^[25]^. In four cases (16.7%), the FET was directly placed into the FL, likely due to shear forces causing tears in the walls of the ascending and descending aorta, which typically positions the FL laterally and the TL medially.

**Figure 2.**
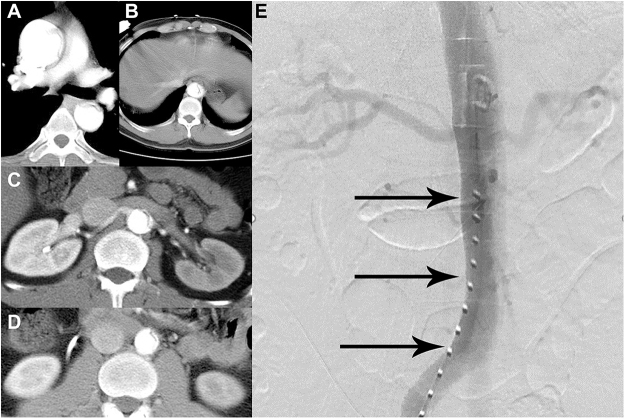
Accidental entry into the FL occurred at the femoral puncture site because the FL-perfused iliac artery was selected by a clinician at our institution. Preoperative CT ((A) ascending aorta, (B) hepatic, (C) pararenal, and (D) infrarenal levels) showing the TL extending along the left anterior side below the renal arteries. Intraoperative angiography (E) revealed that the guidewire had initially entered the FL.

Eleven cases of retrograde TEVAR placement into the FL were reported. In five of these cases (45.5%), accidental guidewire entry into an intimal tear likely caused reentry from the TL into the FL during stent placement. In five cases (45.5%), the cause was unspecified. Notably, one case at our institution involved a TAAD that included both open repair and retrograde FET placement errors. A type II hybrid arch repair was performed with a cardiopulmonary bypass established via the right femoral artery and right atrium. After the aortic root, supra-aortic vessels, and distal ascending aorta were anastomosed, an FET was deployed retrogradely without circulatory arrest. Although intraoperative angiography confirmed correct positioning, the patient developed severe acidosis, hypotension, and elevated pancreatic and liver enzymes. Follow-up computed tomography (CT) suggested the stent was in the FL, prompting emergent reoperation. Preoperative CT findings indicated that the FL supplied the right iliac artery, whereas intraoperative angiography findings indicated that the guidewire had mistakenly punctured into the FL initially. To avoid circulatory arrest, a distal ascending aortic anastomosis was performed with a small stump under distal ascending aortic clamping, which resulted in TL obliteration and allowed the guidewire to remain undetected in the FL throughout the procedure (Fig. 2).

#### Methods for identifying stent misplacement

Among 35 patients, misplacement was detected intraoperatively in 10 (28.6%), detected postoperatively through CT in 15 (42.9%), detected using repeat angiography in 6, and detected using both IVUS and angiography in 1. Three cases (8.6%) were detected retrospectively in postoperative chest X-ray and intraoperative aortography reviews following the patients’ death. Only five patients had intraoperative TEE reconfirmation or IVUS assistance, and all misplacements were detected and corrected intraoperatively. By contrast, among 30 cases eventually assessed using only intraoperative angiography, misplacement was detected in 5 (16.7%) and was undetected in 25. The Pearson chi-squared test indicated that using IVUS or TEE for reconfirmation intraoperatively improved detection (*P* < 0.001; Table 2).

**Table 2:** Intraoperative detection rate

Detect TL/FL method	Angiography alone (*n* = 30)	Combine TEE or IVUS (*n* = 5)
Intra-OP detection number	5	5
Intra-OP detection (%)	16.7%	100%
*P*-value	<0.001	

#### Clinical presentation of misplacement symptoms

Among the 35 cases, mortality was 100% in patients without further intervention and remained at 21.9% in patients who underwent rescue procedures. Moreover, complications were reported in 77.1% of the cases, with only three patients (8.6%) remaining asymptomatic. The most common symptom was visceral malperfusion (17 patients, 48.6%), characterized by acute liver and kidney dysfunction, elevated lactate levels, decreased urine output, and metabolic acidosis. Among these patients, six (35.3%) died – five attributed to multiorgan failure and one to hypertension-related cerebral hemorrhage. Eight patients (22.9%) had unstable hemodynamics, leading to a 37.5% mortality rate (three deaths), with all related to multiorgan failure. Active bleeding occurred in five patients (14.3%), resulting in four deaths (80%), with three attributed to multiorgan failure and one to cerebral hemorrhage. One patient (2.9%) developed permanent paraplegia. Among those who died of acute multiorgan failure, spinal cord ischemia may have occurred but remained undetected or unreported, potentially causing inaccurate assessment of its actual incidence. Moreover, one patient (2.9%) exhibited aortic dilatation at 3 months postoperatively, another had an intraoperative aortic perforation, and three were asymptomatic; all of these patients survived.

Notably, some patients presented with multiple symptoms, which may have potentially affected the outcomes. Our univariate analysis revealed that active bleeding and uncontrollable upper limb hypertension were significantly correlated with increased postoperative mortality (both *P* = 0.002), whereas other symptoms, namely, visceral malperfusion, unstable hemodynamics, aortic dilatation, aortic rupture, and asymptomatic, exhibited no significant correlation. Our multivariate analysis revealed that uncontrollable upper limb hypertension was the only significant predictor of mortality (*P* < 0.001). This discrepancy between the univariate and multivariate findings may be due to confounding and variable interactions among covariates, which potentially diminished the individual effects in the multivariate analysis. Additionally, the limited number of cases and events likely led to reduced statistical power, underscoring the need for caution when interpreting the analytical findings (Table 3).

**Table 3:** Results of univariate and multivariate cox proportional hazards analysis

No. of cases (*n* = 35)	Univariate analysis		Multivariate analysis	
	HR (95% CI)	*P*-value	HR (95% CI)	*P*-value
Intra-OP detection (*n* = 9)	0.607 (0.128–2.869)	0.529		
Symptoms				
Visceral malperfusion (*n* = 8)	2.069 (0.576–7.432)	0.265		
Unstable hemodynamics (*n* = 9)	0.740 (0.373–1.469)	0.390		
Active bleeding (*n* = 5)	0.336 (0.166–0.680)	0.002	0.284 (0.037–2.164)	0.224
Abdominal pain (*n* = 4)	2.831 (0.596–13.445)	0.191		
Upper limb hypertension (*n* = 2)	0.072 (0.013–0.395)	0.002	0.004 (0.000–0.085)	<0.001
Asymptomatic (*n* = 3)	4.980 (0.071–347.241)	0.458		
Aortic dilatation (*n* = 1)	4.632 (0.004–5855.528)	0.674		
Aortic rupture (*n* = 1)	4.590 (0.00–61 712.541)	0.753		
Chest pain (*n* = 1)	0.047 (0.00–74 479.820)	0.674		
Low extremities numbness (*n* = 1)	0.047 (0.00–74 479.820)	0.674		
Paraplegia (*n* = 1)	0.047 (0.00–8 578 676.154)	0.753		
Reintervention method				
Retrograde stent extension with/without septal fenestration (*n* = 22)	0.194 (0.050–0.758)	0.018	8.627 (0.888–83.807)	0.063
Stent graft open removal (*n* = 5)	0.665 (0.084–5.267)	0.700		
Ilio-SMA bypass with septal fenestration (*n* = 1)	5.197 (0.625–43.192)	0.127		
Place additional FET in TL (*n* = 1)	0.047 (0.00–74 479.820)	0.674		
Septal fenestration with SG rerouting (*n* = 1)	5.197 (0.625–43.192)	0.127		
Erroneous elongation of stent graft in FL (*n* = 2)	1.579 (0.199–12.518)	0.665		
Non-intervention (*n* = 3)	14.547 (2.791–75.810)	0.001	0.116 (0.010–1.356)	0.086

### Clinical interventions and outcomes

#### Management strategies

##### Septal fenestration with stent graft elongation

Among the 32 intervention cases, 22 (68.8%) were managed with redirection and endovascular stent elongation. Among these, 19 involved endovascular septal fenestration. Three cases involved extending the stent graft from the FL through an intimal initial entry to preserve distal organ perfusion – two below the previously misplaced stent and the other near the celiac trunk orifice.

In the absence of a suitable intimal initial entry, various practicable techniques for creating an artificial tear have been described – such as endovascular approaches, including the use of a 0.014-inch Grand Slam guidewire (Abbott Vascular, Abbott Park, IL, USA), for puncturing the septum^[26]^. Raupach *et al* noted the benefits of a subintimal reentry catheter (Outback Elite, Cordis, Miami, FL, USA), which is less costly and requires less training to use^[5]^. Other methods involve radiofrequency ablation with the TourGuide (Medtronic, Minneapolis, MN) articulating sheath and PowerWire (Baylis Medical Company) device or a combination of radiofrequency ablation with downward traction to make a big, nonrestricting slit fenestration^[25,27,28]^. Transseptal needles, including transjugular intrahepatic portosystemic shunt needles and reentry devices, have also been utilized^[5,29]^. Reentry devices are considered safer owing to the shorter needles involved, which minimize the aortic wall injury risk^[29]^. The use of electrocautery applied to a short, circumferentially denuded guidewire has been reported to enable controlled dissection flap fenestration, thereby optimizing both proximal and distal landing zones, creating a common channel, and facilitating the deployment of branched or fenestrated endograft. This technique represents a feasible and promising strategy for managing visceral and lower-limb malperfusion in complex aortic repair^[30]^. Additionally, balloon inflation in the opposite lumen can stabilize the septum for puncture but carries risks, such as embolism from balloon fragments or air^[29]^. Open surgical methods to fenestrate the intimal flap have also been reported^[31]^.

Regarding the recommended site of septal fenestration, it should be located 10–40 mm distal to the previously placed stent graft. This spacing allows for additional stent graft placement while minimizing the risk of compromising blood flow to the visceral arteries^[26,32–35]^. Moreover, case reports have demonstrated that newly placed stent grafts often overlap with existing grafts, with approximately half in the FL and half in the TL^[26,35,36]^.

##### Open technique for stent graft removal

An open technique was used in five cases (14.3%). In four cases (80%), open repair through median sternotomy, with or without anterolateral thoracotomy, was performed to remove the malpositioned FET^[37–40]^. The ascending aorta was cross-clamped, and the supra-aortic vessels were dissected, revealing the stent graft in the FL. In three cases, a new FET was deployed, and the arch was reconstructed with a four-branch Dacron prosthesis. Additionally, in one case, reconstruction details were unspecified. In the final case, the misplaced FET was promptly removed intraoperatively without redoing circulatory arrest postoperatively.

In one case, an extra-anatomical bypass and septal fenestration were performed to treat malposition and bowel ischemia. A bypass from the right external iliac to the superior mesenteric artery (SMA) was created, with fenestration of the abdominal aorta to improve TL perfusion. Despite these efforts, the patient died of multiorgan failure on postoperative day 7^[6]^.

##### Other methods

Additional interventions included the placement of a longer FET in the TL to compress the misplaced stent and ilio-SMA bypass with aortic fenestration; three patients received no intervention. We also noted the misidentification of the TL and FL during correction in two cases, leading to stent graft extension within the FL.

#### Management outcomes

Regarding patient outcomes, 24 (68.6%) survived, 10 (28.6%) died, and 1 case was unreported. Among 22 patients who underwent endovascular retrograde stent extension with or without septal fenestration, 19 (86.4%) survived. The patient with the stent extension near the celiac trunk died. Among four patients who underwent thoracic stent removal with aortic arch replacement, three (75%) survived. The patient with prompt intraoperative FET removal survived. All three patients without active intervention died within 2–5 days. One of two patients with erroneous FL extensions survived because of sufficient reentry perfusion into the TL.

In the univariate analysis, nonintervention cases had significantly higher mortality (*P* = 0.001). Among the methods examined, only endovascular stent graft elongation with or without septal fenestration was associated with a significantly higher survival rate (*P* = 0.018). Although extra-anatomical bypass had a 100% mortality rate, this finding could not be generalized because of the small sample size (*n* = 1). These findings suggest that endovascular repair is the preferred approach, whereas high mortality in extra-anatomical bypass may be related to late detection, reintervention, and prolonged ischemia duration and severity.

#### Time to treatment and its influence on survival

**Figure 3.**
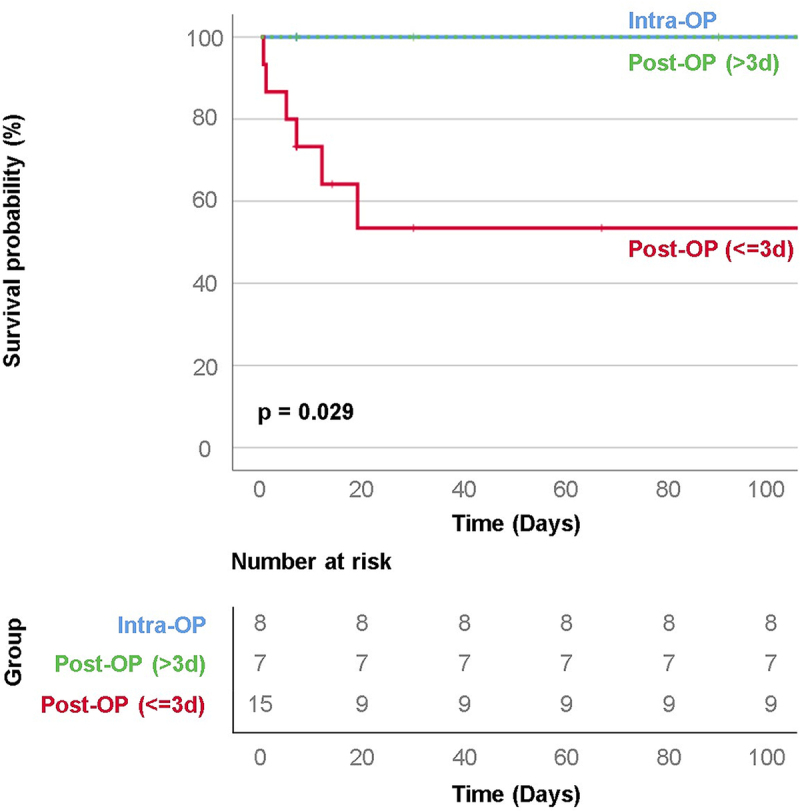
Kaplan–Meier curve for patients categorized by intervention timing: intraoperatively, within 3 days, and more than 3 days postoperatively.

The patients were grouped by intervention timing: intraoperatively, within 3 days postoperatively, and 3 days or more postoperatively (excluding nonintervention and FL stent elongation cases). Among 15 patients treated within 3 days, 6 (40%) died – 5 attributed to multiorgan failure and 1 attributed to intracerebral hemorrhage. No deaths occurred among those treated intraoperatively or after 3 days. Our Kaplan–Meier analysis revealed significantly higher mortality rates for cases managed within 3 days (*P* = 0.029), suggesting better outcomes with intraoperative management or delayed symptom presentation. By contrast, mortality significantly increased when symptoms developed within 3 days. This phenomenon may be attributed to the presence of sufficient reentry sites in delayed-symptom cases, which can reduce acute hemodynamic compromise, delay deterioration, and improve prognosis (Fig. 3).

#### Consequences of erroneous stent graft elongation in the FL

**Figure 4.**
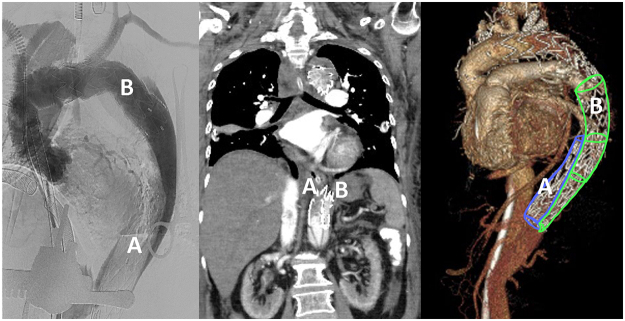
Angiography occasionally led to misidentified stent positions, suggesting stent A in the FL and stent B in the TL, which led to stent B being extended during surgery. However, postoperative CT revealed the error (middle panel), significantly aiding decision-making and improving patient outcomes.

In our review, two cases involved erroneous stent graft elongation into the FL. In the first case, preoperative CT indicated the TL to be in the medial position of the descending aorta, and intraoperative aortography confirmed the location of the TL during FET placement. After the antegrade deployment of two FET stents, angiography findings revealed distal migration of the first stent, suggesting its likely placement into the FL. An attempt to extend the second stent and compress the first with a balloon to alleviate significant suture-line bleeding proved in vain, requiring over 60 units of blood transfusion. Postoperative CT confirmed that the first stent was located in the TL and that the second stent had its proximal end in the TL but its distal end in the FL, indicating the accidental extension of the FL (Fig. 4). Despite extracorporeal membrane oxygenation (ECMO) support, the patient died of intracranial hemorrhage and multiorgan failure on postoperative day 11. In the second case, after FET placement, an enlarging aortic dissecting aneurysm with a suspected type II endoleak developed. Four thoracic grafts wrongly extended the stent into the FL, and the celiac trunk was coiled to secure the distal landing zone. Fortunately, distal perfusion was maintained by several intimal reentry sites, and the patient remained stable until requiring intervention for an 8-cm thoracoabdominal aneurysm 11 years later.

#### Left ventricular failure and ECMO utilization

Studies have yet to report the association between FET misplacement, increased afterload, acute left ventricular failure, and ECMO utilization. At our institution, two of six cases with postoperative cardiac dysfunction, initially considered to be due to poor myocardial protection during surgery, required ECMO. Although peripheral-type ECMO reduces cardiac load and enhances visceral TL perfusion, it can mask FET misplacement and delay diagnosis. Accordingly, in cases of postoperative unexplainable acute heart failure without perioperative coronary events after FET placement, clinicians should consider the possibility of misplacement before peripheral-type ECMO to ensure timely intervention.

### Quality assessment

A standardized assessment tool was used to evaluate the quality of evidence for the 35 cases. Of the studies, 3 (8.6%) had a high risk of bias, 7 (20%) had a moderate risk of bias, and 25 (71.4%) had a low risk of bias. Stent graft misplacement could not be confirmed in three cases (8.6%) because of missing postoperative CT scans in two cases and delayed diagnosis in one. Seven cases (20%) had inadequate descriptions of misplacement symptoms, and 12 (34.3%) lacked sufficient follow-up for outcomes to be assessed fully. Reporting was insufficient for replication or clinical practice in 12 cases (34.3%). Details on the quality assessment process, including adherence to the CARE guidelines, are provided in Supplemental Digital Content Table S3, available at: http://links.lww.com/JS9/E775, and Supplemental Digital Content Table S4, available at: http://links.lww.com/JS9/E776.

## Discussion

### Importance of this systematic review

Although rare, accidental TEVAR stent graft placement into the FL is a clinically significant event that may lead to severe complications during the treatment of aortic dissection or aneurysm. Misplaced stents cause FL perfusion, worsening the dissection, increasing FL pressure and left heart afterload, and leading to severe outcomes such as intracranial hypertension and organ malperfusion. To date, most published case reports have focused on intraoperative management, with limited emphasis on procedural patterns or the timing of FL deployment. The lack of pooled data on timing and outcomes has restricted our understanding of TEVAR misplacement. Further research is warranted to clarify the causes of misplacement and assess the efficacy of various interventional strategies. Therefore, this systematic review is the first to analyze 35 reported cases.

According to our review, only 29 cases have been reported in the past 40 years. However, we observed that several young patients with normal coronary arteries, intact valves, and only brief cardiac arrest rapidly died of acute heart failure postoperatively before follow-up CT could be performed. Despite the absence of CT scan results confirming stent misplacement, a retrospective review of preoperative and postoperative radiographs, coupled with the absence of other alternative causes, suggested that stent misplacement could be a strong possibility. Nevertheless, misplacement was not suspected by the surgical team at the time, and identification and subsequent reporting were unlikely. Therefore, considering the diagnostic difficulty and potential underreporting, current data may not fully capture the true incidence of TEVAR misplacement.

By synthesizing diverse case reports and correlating treatment delays with survival, this review provides preliminary insights into prognostic factors. Although the therapeutic approaches varied among the included studies, we propose a tentative unifying intervention framework that may assist clinicians in making decisions and selecting management strategies. Our findings also demonstrate that sole reliance on angiography may occasionally lead to interpretive errors. Early recognition, timely management, and preventive methods are essential to minimize the risk of these adverse events and improve patient outcomes.

### Strategies for reducing the risk of misplacement

#### Precise preoperative CT evaluation

Accidental FET placement into the FL is potentially preventable. Accurate preoperative and intraoperative identification of the TL and FL is fundamental to ensuring appropriate placement. Considering the considerable variation in the spatial relationship between the TL and FL, misinterpretation remains a risk. Therefore, a comprehensive preoperative CT analysis, including mapping the whole pathway of both lumens and locating intimal tears by size, number, and position, is essential. In four reported cases, the FET was deployed directly into the FL under direct vision, likely due to inadequate preoperative delineation of the TL pathway. These findings also highlight that in certain situations, a medially positioned, smooth-lining, thrombus-free FL can be mistaken for the TL during operation, particularly when the TL is presumed to be medial and inner, underscoring the need for meticulous preoperative planning.

#### Antegrade placement

Antegrade placement is commonly utilized in hybrid procedures and is particularly advantageous when retrograde access is limited by severe peripheral artery disease or a tortuous aorta, which complicates femoral or iliac vessel guidewire passage^[41–43]^. When the antegrade approach is used, the risk of distal stent graft misplacement into the FL due to reentry, iatrogenic intimal tears, or direct FET introduction without visual confirmation of the distal landing zone can be minimized by presetting a retrograde guidewire^[6,13]^. The guidewire should pass through the TL, with its position repeatedly confirmed using segmental aortography, and should enter the aortic arch via the femoral artery, particularly when the entry point is preoperatively identified at the proximal descending aorta^[18]^. However, accidental entry into the FL at the femoral puncture site may occur if the clinician chooses the wrong FL-perfused iliac artery side, as was the case in our institution (Fig. 2). Additionally, selecting the correct stent graft length is crucial. Preoperative enhanced CT should measure the distance from the left subclavian artery to the entry point for effective sealing^[6]^.

#### Retrograde placement

Retrograde placement is less invasive than open surgery and allows for rapid femoral access, rendering it an ideal option for emergency procedures. Consistent with our study, several studies have recommended using TEE or IVUS reconfirmation intraoperatively to reduce the risk of retrograde FET misplacement^[18]^ through more timely detection (*P* < 0.001). A three-dimensional virtual intravascular endoscopy may also be a viable alternative. Angiography alone may fail to distinguish the TL from the FL; this is because contrast agents can reenter the TL through reentry sites, leading to misinterpretation (Fig. 4).

Routine use of IVUS or TEE adds cost and requires additional personnel; we recommend their use when acute heart failure, massive bleeding, visceral malperfusion, or other symptoms mentioned in this review occur. Nevertheless, in the absence of operator awareness or experience, intraoperative TEE may result in false negative findings in rare cases^[6]^. Therefore, monitoring the pressure deficit of upper and lower limb artery pressures, both intra- and post-operatively, is also recommended^[6]^. Recognizing misplacement symptoms and considering them in differential diagnoses are also crucial to prevent adverse outcomes.

#### Framework for preventing FL misplacement

**Figure 5.**
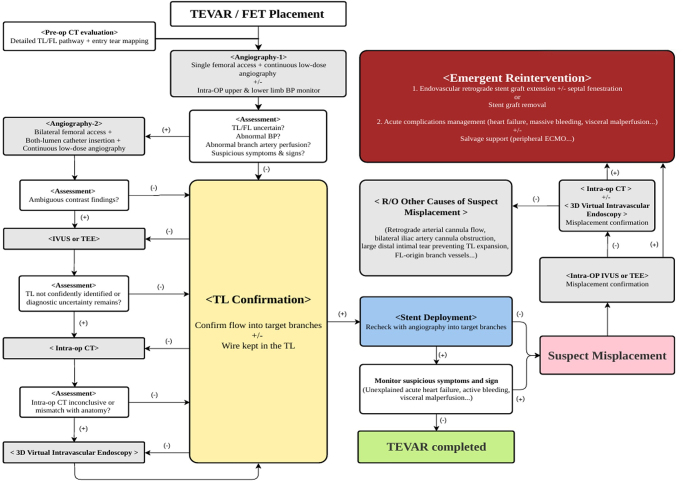
Proposed workflow to aid decision-making in intraoperative imaging in TEVAR or FET procedures, particularly in complex or high-risk cases.

We propose a workflow that incorporates intraoperative adjuncts for TEVAR and accounts for both diagnostic utility and economic feasibility (Fig. 5). This preliminary framework may aid in refining procedural protocols and imaging strategies in future TEVAR or FET interventions. Although further validation is required, this approach may reduce the risk of FL misplacement and improve procedural safety. Considering the rarity and severity of this complication, prospective or randomized studies are unlikely to be feasible. Therefore, additional comparative studies of intraoperative imaging modalities (which focus on assessing cost-effectiveness, diagnostic accuracy, and clinical applicability), along with consensus-based guidelines, may help in the identification of optimal strategies for the early detection and prevention of such complications.

### Limitations

This is the first systematic review of TEVAR misplacement into the FL, and its strength lies in its systematic approach and standardized quality assessment for the available case reports. However, it has several limitations. Case reports are anecdotal and nonrandomized, limiting causal inference. The review relied on nonsystematic clinical data, often lacking details due to publication constraints, which may have introduced bias. Furthermore, because case reports represent the lowest tier of clinical evidence, the overall quality of evidence was typically low. Although the survival rate observed among the 35 cases was 68.6%, the actual survival rate might be lower because 12 of the cases did not have sufficient follow-up data beyond 1 month. Previous studies have reported a 30-day mortality of up to 66%^[4]^, with 83.3% at our institution, suggesting the initial rate may not reflect the actual mortality. Additionally, underreporting of this complication further limits statistical power. Despite these issues, the study’s strength lies in its systematic approach and standardized quality assessment for the available case reports.

## Conclusions

Accidental TEVAR stent graft placement in the FL, although rare, is underreported and potentially catastrophic but treatable and preventable. Despite seemingly acceptable survival rates, challenges in diagnosis and the likelihood of publication bias may lead to misleading results. Misplacement may occur if the stent graft is placed without a guidewire or pigtail catheter in the repeatedly confirmed TL. Active bleeding and uncontrollable upper limb hypertension due to afterload escalation indicate a high mortality risk, warranting prompt diagnosis. Intraoperative IVUS or TEE reconfirmation can enhance early detection; this is because undiagnosed cases worsen if symptoms arise within 3 days. Regarding management, endovascular retrograde stent extension with or without septal fenestration is the most effective treatment strategy. Finally, comprehensive aortography is also vital for early diagnosis and intervention.

## Data Availability

The datasets generated during the current study are available from the corresponding author on reasonable request.

## Research registration unique identifying number (UIN)

This study was registered at PROSPERO (Registration Number: CRD42025645867): https://www.crd.york.ac.uk/PROSPERO/view/CRD42025645867.
